# Perceptions of the critical cultural competence of registered nurses in Canada

**DOI:** 10.1186/s12912-017-0242-2

**Published:** 2017-08-15

**Authors:** Adel F. Almutairi, Abdallah A. Adlan, Maliha Nasim

**Affiliations:** 1 King Abdullah International Medical Research Center (KAIMRC), Riyadh, Saudi Arabia; 2 King Saud Bin Abdulaziz University of Health Sciences, Ministry of National Guard – Health Affairs, Riyadh, Saudi Arabia; 3 University of British Columbia, Vancouver, Canada

**Keywords:** Cultural diversity, Cultural competence, Multicultural workforce, Rregistered nursing, Canada

## Abstract

**Background:**

Cultural diversity often leads to misunderstandings, clashes, conflicts, ethnocentrism, discrimination, and stereotyping due to the frequent intersection of many variables, such as differences in traditions, behaviours, ethical and moral perspectives, conceptions of health and illness, and language barriers. The root of the issue is related to the way people conceptualise differences and the unique cultural and historical circumstances that have shaped different groups’ heritages. In this study, therefore, we aimed to investigate the perceptions of critical cultural competence (CCC) of registered nurses working in various hospitals across the province of British Columbia, Canada.

**Method:**

Data were collected using Almutairi’s Critical Cultural Competence Scale (CCC Scale) with a random sample of 170 registered nurses. This scale measures four essential multidimensional components of the CCC model: critical awareness, critical knowledge, critical skills, and critical empowerment. Data were analysed using descriptive and inferential statistics (Kruskal-Wallis test).

**Results:**

The data revealed that participants’ perceptions of CCC were positive with a mean score of 5.22 out of 7.00 for the total number of items (*n* = 43) and a standard deviation of 0.54. The mean scores for the CCC subscales ranged from 4.76 (for critical skills) to 5.42 (for critical empowerment). The results indicated a statistical difference in CCC perceptions based on participants’ age and country of birth with *p* = 0.05 < 0.05 and 0.029 < 0.05, respectively.

**Conclusion:**

Nurses’ age (experience) and country of birth may influence their perceptions of CCC as gaining cultural competence requires exposure to caring for patients from various cultures and countries, and is associated with cultural knowledge and awareness. Therefore, this finding reveals that healthcare organizations must provide ongoing cultural education programs to increase their nursing staff’s level of cultural competence so they are better able to deal with the difficulties that might arise during cross-cultural interactions.

## Background

The concept of cultural diversity—in a multicultural environment—refers to the existence of a number of cultures that operate simultaneously in one space. Such diversity is a result of many factors, including colonisation, forced or voluntary immigration, acculturation, mobility of workforces, and so forth [1, 2]. People from different cultures bring with them their own beliefs, values, traditions, behaviours, ethical and moral perspectives, and languages, as well as unique and individual historical, political, and economic circumstances. Such complex differences can lead to misunderstandings, clashes, conflicts, negative attitudes, ethnocentrism, discrimination, and stereotyping due to the frequent intersection of these variables [3]. Such complexity can negatively impact the health and well-being of all involved members.

Many studies—conducted in different contexts and with different populations—indicated that the profound consequences of cultural and linguistic diversity in healthcare can lead to healthcare inequities, disparities, and put the physical, mental, spiritual, and social safety of people at risk [3–8]. The literature concerning cultural diversity engenders a feeling that such diversity is often blamed for complexities within a multicultural healthcare environment. This is certainly not the case as cultures, in general, are valuable heritages that enrich societies and provide lenses through which to view and interpret the realities around us from multiple perspectives. The problem, however, lies in the way people conceptualise complex layers of differences in other cultures and their level of preparedness to deal with them.

**Fig. 1:**
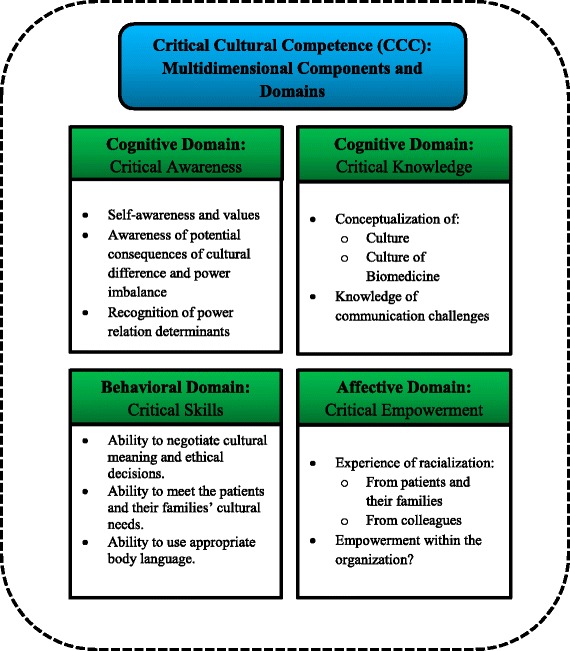
Multifaceted components of Critical Cultural Competence (CCC)

Such ongoing complexities, therefore, have prompted many scholars in this field to develop theoretical frameworks in an attempt to manage the difficulties and challenges that healthcare providers often encounter during cross-cultural interactions [9–12]. These difficulties can be experienced among healthcare providers themselves or between them and their patients. One influential and comprehensive theoretical framework, which can be applied in different contexts across the world, is Almutairi’s Critical Cultural Competence (CCC) [13]. The CCC is a process described as “a lifelong learning endeavour that requires individual agency, and policy guidance that addresses the sociocultural determinants of health, and support at the organizational level” (13, p. 3). This framework is composed of four major and multifaceted components: critical awareness, critical knowledge, critical skills, and critical empowerment. Each of these components represents a distinct conceptual domain, as illustrated in Fig. 1.

The purpose of this study, therefore, was to investigate the perceptions of critical cultural competence of registered nurses working in various hospitals, across the province of British Columbia, Canada, using the Critical Cultural Competence Scale.

## Methods

### Population, sampling, and recruitment

The target population of this study was the registered nurses who are currently working in various hospitals in the province of British Columbia. These nurses were selected randomly based on the following criteria: (a) registered with the College of Registered Nurses of British Columbia (CRNBC), (b) agreed to be contacted for research purposes, and (c) holding practicing registration and working either full time or part time. Therefore, the approach to recruiting the nurses was through the CRNBC, which has the addresses of all registered nurses working in the province and who are willing to participate in research activities. The stamped recruitment and data collection packages were provided to the CRNBC to be sent to the randomly (computer generated), eligible nurses (*N* = 1000). The data collection package contained an invitation letter, a cover sheet explaining the study’s purpose and procedure, and the research team members’ contact details as well as a prepaid envelope to return the completed instruments. Participants were asked to seal the survey in the enclosed, self-addressed and stamped envelope and to mail it to the researcher within 3 weeks of receiving it. The return of the completed survey was considered to be consent for participation. The expected time for completing the survey was approximately 30 min.

### Data collection

A critical cultural competence (CCC) scale was used to measure the registered nurses’ perceptions of critical cultural competence in a multicultural context. This scale was originally developed by Almutairi, Dahinten and Rodney in 2013, through a systematic development process and evaluation procedures as indicated in their published papers [14, 15]. The CCC scale comprised 43 items that measure four essential multidimensional components of the CCC model: critical awareness, critical knowledge, critical skill, and critical empowerment. Participants were asked to rate their views on a 7-point Likert scale, anchored from “strongly disagree” to “strongly agree,” and “never” to “always.” Such a scale can determine the participants’ levels of agreement with the statements. Participants needed to obtain a mean score of five or above in order for their perceptions of CCC to be considered positive.

The psychometric properties of the CCC scale were thoroughly investigated by Almutairi, and Dahinten [14, 15] using different types of analysis statistics such as factor analysis and convergent and discriminant validity. Their studies have yielded strong empirical evidence for the construct validity and reliability of the CCC scale. Cronbach’s alpha coefficient for the total scale scores was adequate (0.86).

In addition, the survey comprised a section to obtain the demographic information of the respondents, which included their age in years, job position, educational level, cultural training, years of work experience, work specialty or department, racial/ethnic background, and country of birth.

### Data analysis

Data analysis was performed using the Statistical Package for the Social Sciences, version 20 (SPSS). The characteristics of the study sample were measured using descriptive statistics. For example, frequency was used to analyse the participants’ demographic data; on the other hand, mean and standard deviation were used to analyse the continuous data. Inferential statistics were used to examine the differences between groups in terms of their perceptions of CCC. These groups were categorised according to age, educational level, cultural training, years of experience, and racial/ethnic background.

In order to identify the appropriate statistical tests for such analysis, the assumption of normality of the current dataset was evaluated. For example, normal distribution of the data dictates using parametric statistics; otherwise, nonparametric statistics are the best choice. Therefore, the Kolmogorov-Smirnov test was conducted, which is used to assess the distributions of the scores. The outcome of this test suggests normal distribution if *p* > .05; however, *p* < .05 indicates a violation of the normality assumption [16]. In this study, the *p* value was .049, indicating that the data were not normally distributed. This violation of the normality assumption was verified by inspection of the histogram. Therefore, nonparametric statistics were used in this study, namely the Kruskal-Wallis test, which is analogous to the One Way Analysis of Variance (ANOVA).

### Ethical considerations

Prior to undertaking this study, ethical approval was obtained from the Behavioural Research Ethics Board in the University of British Columbia with reference number (H13–02247).

## Results

### Response rate

Although incentive was provided for participants, this study received a poor response rate. Specifically, the study packages were sent to 1000 registered nurses across the province of British Colombia, and only 170 nurses completed and returned the instrument. Such a poor response was expected as this study was part of a larger study that involves five other instruments. The sample size was, however, sufficient for the correlational analysis.

### Participants’ profile

Females made up the majority of participants (*N* = 154, 90.6%) in this sample, whereas the number of male participants was only 14 (8.2%). Participants’ ages ranged from 23 to 67 years old (M = 43.67; SD = 11.8). The age of the majority of participants (31.3%) ranged from 50 to 59 years, followed by those between 30 to 39 years old (23.6%). In addition, the majority of participants (*N* = 99, 59%) possessed a bachelor of science in nursing degree, and the remainder of the sample had either a diploma of nursing (*N* = 60, 35.7%) or a master’s degrees (*N* = 9, 5.4%). The job positions of the participants varied; however, the great majority worked as staff nurses (*N* = 149, 88.7%). The rest of the sample (11.4%) worked in various positions, including nurse practitioner, case manager, home-care nurse, and supervisor. The nurses had varying lengths of experience, either in their current organization (M = 11.14; SD = 9.5) or in their profession (M = 16.60; SD = 12.14). Only 38.8% of participants (*N* = 66) had had any form of cultural training some time during their career.

The majority of the participants described themselves as Caucasian (*N* = 126, 75.4%), while the second largest group identified as South Asian (*N* = 20, 12%); then Chinese (*N* = 9, 5.3%), Far East Asian (*N* = 3, 1.8%), other Asian (*N* = 2, 1.2%), and First Nation (*N* = 1, 0.6%). In addition, six of the participants (3.6%) described themselves as a mixture of ethnic backgrounds, such as First Nation/Caucasian, Japanese/Caucasian, and Eurasian. Participants stated that they were born in different countries (*N* = 23) including Canada, the Philippines, Taiwan, Hong Kong, South Korea, Slovakia, China, Ireland, Germany, Netherlands, Paraguay, Hungary, the United Kingdom, India, Russia, the United States, Zimbabwe, South Africa, Ukraine, Australia, Japan, Iran, and Jamaica.

### Nurses’ perceptions of CCC

The mean score for the total number of items (*n* = 43) and respondents (*N* = 170) was 5.22 out of 7.00, with standard deviation of 0.54. A higher mean score (M > 5) indicates a positive perception of CCC. The mean scores for the CCC subscales ranged from 4.76 (for critical skills) to 5.42 (for critical empowerment). Table 1 shows the mean scores and standard deviation for these scales.

**Table 1:** The mean scores and standard deviation of the CCC scale and subscales

Scale	N	Minimum	Maximum	Mean	Std. Deviation
Critical Awareness	170	3.08	6.23	5.0461	.57553
Critical Knowledge	166	3.00	6.83	5.3504	.83538
Critical Skills	170	2.29	7.00	4.7629	.86781
Critical Empowerment	170	1.86	6.95	5.4246	.97213
CCC Scale	170	3.20	6.20	5.2229	.53870
Valid N (listwise)	166				

As can be seen in Table 1 above, the mean score and SD for the critical awareness subscale were 5.04 and 0.57, respectively, which is within the positive range. The negatively worded statement, “When I interact with people from other cultures, I feel my cultural values and norms are better,” had the highest positive mean (5.69). The vast majority of respondents (*N* = 60, 35.3%) strongly disagreed with this statement, 26.5% (45) disagreed, and 17.8% (*N* = 30) slightly disagreed; whereas, 17.1% (*N* = 29) of respondents either slightly agreed or agreed. In contrast, the statement that has the lowest mean was “I find it annoying when time is not important for some people from other cultures.” The majority of participants (38%, *N* = 64) indicated that they occasionally have this feeling, 24% indicated that they have it frequently, and 30% rarely.

The critical skills subscale had the lowest mean score of 4.76 and SD 0.86, which is still close to the positive score. The lowest scored statement was “I discuss ethically sensitive issues with my patients/families” with a mean score of 3.93. About 32.9% of respondents indicated that they rarely to never discuss ethical issues with their patients, while 35.9% indicated that they occasionally do. Participants also had diverse views about discussing the different cultural meanings of health and illness with their patients from other cultures in an attempt to provide them with optimal care. Of the participants, 34.7% (*N* = 59) reported occasional discussion of different cultural perspectives with their patients, while 31.8% (*N* = 52) either rarely or never did. Table 2 presented the means and standard deviations of participants’ responses to individual items.

**Table 2:** The means and standard deviations of the responses on the items

#	Items	N	Mean	SD
Critical Awareness				
1.	Gender can determine the way people relate to others.	168	5.26	1.268
2.	Social class is an important factor in determining the way people relate to others.	168	4.56	1.558
3.	Gender roles, positions or activities can empower some people over others.	168	5.43	1.207
4.	Cultural differences between people can generate conflicts and tensions during interactions.	167	5.67	1.154
5.	Cultural differences influence how we interact and relate to others from other cultures.	166	4.96	1.288
6.	An interpersonal power imbalance could compromise a healthcare provider’s well-being.	167	5.38	1.230
7.	When I interact with people from other cultures, I feel my cultural values and norms are better.	167	5.69	1.366
8.	I find it annoying when time is not important for some people from other cultures.	167	4.34	1.413
9.	The large number of visitors for patients from other cultures is a nuisance.	167	4.66	1.374
10.	Cultural and linguistic differences could compromise healthcare provider’s well-being.	168	4.86	1.598
11.	In a multicultural environment, ‘race’ can determine the way people relate to others.	168	4.88	1.299
12.	Cultural and linguistic differences between the healthcare provider and patient could compromise patient’s safety.	168	5.42	1.573
Critical Knowledge				
13.	There are no cultural variations between different cultural groups. [R]	166	6.31	.845
14.	There are no cultural variations within a cultural group of people. [R]	166	6.21	.971
15.	It is not important to assess a patient’s preferences in terms of healthcare services if I am knowledgeable about their culture. [R]	166	5.70	1.519
16.	People from the same culture have the same religion.	169	6.08	.978
17.	Western biomedicine is always attentive to diverse cultural meanings.	166	5.02	1.392
18.	Cultural norms that people adhere to are largely fixed and unvarying. [R]	166	4.71	1.444
19.	It is easy to anticipate behaviors and practices of people if I know their culture. [R]	166	4.14	1.600
Critical Skills				
20.	I use some of my patients’ languages during my care if I know a little.	166	4.62	1.739
21.	I use culturally congruent body language when interacting with people from other cultures.	164	4.49	1.572
22.	I am able to change healthcare practices to meet my patients’ cultural and religious needs and expectations.	165	4.38	1.285
23.	I discuss the different cultural meanings in terms of health and illness with my patients from other cultures in order to provide them with optimal care.	166	4.03	1.394
24.	I use simple language when I speak with people from other cultures and I consider their potential language limitations.	165	5.52	1.167
25.	I discuss ethically sensitive issues with my patients/families.	166	3.93	1.324
26.	It is not important to assess a patient’s preferences in terms of healthcare services if I am knowledgeable about their culture.	165	6.38	.768
Critical Empowerment				
27.	I feel disrespected because of my culture. [R]	167	6.01	1.382
28.	I face biased remarks and racism. [R]	167	5.71	1.640
29.	I feel alienated in my workplace because of my cultural background. [R]	166	6.15	1.239
30.	I am treated differently to my counterparts by management because of my cultural background. [R]	167	5.73	1.458
31.	I feel disrespected because of my gender.	167	5.60	1.679
32.	In this organization, people are treated differently according to their country of origin. [R]	167	5.37	1.573
33.	I have an equal opportunity in terms of professional development compared to colleagues in my organization.	167	5.29	1.470
34.	My colleagues from other cultures look down on me. [R]	166	6.33	1.086
35.	I feel that my colleagues from other cultures are more powerful. [R]	168	5.47	1.484
36.	I feel that my skin colour determines how people relate to me in this context. [R]	167	5.25	1.741
37.	I have an equal opportunity in terms of promotion compared to colleagues in my organization.	167	5.12	1.536
38.	I worry about losing my job if I speak out about my concerns of discrimination. [R]	167	5.14	1.697
39.	I feel reluctant to speak out about my conditions of work. [R]	166	5.37	1.630
40.	CCC46: My nursing competence is frequently challenged by my colleagues compared to that of my counterparts. [R]	167	5.86	1.303
41.	I feel safe in expressing my concerns to management.	167	4.77	1.679
42.	My nursing competence is frequently challenged by my patients compared to that of my counterparts. [R]	167	5.80	1.345
43.	Social norms limit my ability to advocate for my patients. [R]	168	5.05	1.529

### Comparisons between groups based on

#### Age

Non-parametric testing, namely, the Kruskal-Wallis test, of K-independent samples was used to examine the differences between nurses’ perceptions of CCC based on their age groups to identify the potential relationships between these two variables. The results indicated that there is a statistical significance in CCC across the different age groups of participants with a significance level of *p* = 0.005 < 0.05 and chi-square H = 14.983. Inspection of the mean rank, which describes the direction of difference, indicated that the group aged 30 to 34 has the highest perception of CCC, while the group aged 35 to 39 has the lowest perception of CCC, as shown in Table 3 below.

**Table 3:** Difference in nurses’ perceptions of CCC based on age groups

Part A		
Age in years	N	CCC Mean Rank
Less than30	27	71.17
30–34	25	99.30
35–39	15	47.37
40–44	17	81.29
45 years and over	84	91.66
Total	168	
Part B		
Test statistics^a, ﻿b^		
	CCCS	
Chi-square	14.983	
Df	4	
Asymp. sig.	.005	

Note:

^a^Kruskal Wallis Test

^b^Grouping variable: age in years

#### Country of birth

The same testing technique (Kruskal-Wallis) was conducted to determine the differences in CCC perceptions between groups based on their country of birth. As described earlier, nurses stated that they were born in different countries across the world including Canada. Discounting minor differences in culture and traditions, these countries were combined to form three major categories: Anglo-Saxon countries, European countries, and Asian countries. The grouping was imperative due to the very limited number of respondents from these countries and was not statistically significant enough to run correlation tests between groups based on their country of birth. Three countries were excluded, namely, Zimbabwe, South Africa, and Jamaica, because only one participant was born in each country and, consequently, they could not form a separate group. The result of the test indicated that there is a statistical difference in CCC perceptions based on participants’ countries of birth with a *p* value of 0.029 < 0.05, H = 14.983. The mean ranks in Table 4 indicated that nurses who were born in Anglo-Saxon countries had the highest level of CCC perceptions, and nurses who indicated that they were born in Asian countries had the lowest perceptions.

**Table 4:** Difference in nurses’ perception of CCC based on country of birth

Part 1		
Country of birth (Groups)	N	Mean Rank
Anglo-Saxon countries	122	88.27
European countries	15	79.77
Asian countries	28	61.79
Total	165	
Part 2		
Test statistics^a, b^		
	CCCS	
Chi-square	7.074	
df	2	
Asymp. sig.	.029	

Note:

^a^Kruskal Wallis Test

^b^Grouping variable: country of birth

## Discussion

Our study found significant differences in nurses’ perceptions of CCC based on age group, as explained earlier in the results section (Table 3). Interestingly, nurses within the 30–34 years age category reported the highest scores for CCC, followed by nurses over 45 years and then those from 40 to 44 years. The lowest scores were found among nurses aged 35–39. This finding is almost similar to the findings reported by Bunjitpomol and her colleagues in that knowledge of cultural competence is positively associated with age and responsibility [17]. In the same line, another study investigated cultural competence among nurses in Israel, found a positive relationship between cultural skills and age/years of experience [18]. Although our finding demonstrated the relationship between age/years of experience and perception of critical cultural competence, there are many factors could influence nurses’ perceptions of CCC, and result in perceptions differences between age groups. For example cultural encounter or interaction and effective communication play an important role in the process of becoming culturally competent [12], which will allow nurses to engage and understand the cultural needs of patients from diverse cultural backgrounds.

In addition, the formal integration of transcultural education in the university curriculum may have benefited younger nurses (under the age of 30 years) to have high perceptions of cultural competence. Studies have highlighted the benefits of early transcultural education and diversity training in increasing the level of cultural competence [19–21]. Furthermore, the higher scores observed in older nurses (40+ years) in our study may reflect a greater number of years of work experience [20], as well as more participation in transcultural educational training [22–25] and more time caring for patients from diverse ethnicities. These factors have been found in numerous studies to predict and correlate with better cultural competence [26–28].

Our findings showed that study participants born in Anglo-Saxon countries (US, UK, Australia, NZ & Canada) had the highest mean CCC scores, followed by those born in European countries. In contrast, nurses born in Asian countries reported the lowest CCC score (Table 4). One explanation for this result may be that Anglo-Saxon nurses share a similar cultural background as the majority of their patients. More than 75% of our sample described themselves as Caucasian, and according to statistics taken from census data, the proportional composition of British Columbia is heavily weighted in favour of ethnic groups from Anglo-Saxon and European countries [29]. Assuming the majority of patients’ ethnic backgrounds mirror those of the population at large, nurses and patients are likely to share the same language and cultural characteristics, which may explain the higher CCC scores reported. A similar study assessing cultural competence among South African ICU nurses supports our results [30]. Their findings reported significantly higher cultural competence scores among nurses who spoke a second language at home (not English, but mainly the isiZulu language) compared to nurses who spoke English. Non-English speaking nurses had significantly higher scores for cultural encounters, cultural desire and cultural awareness compared to English speaking nurses. The ability for patients and nurses to communicate through a shared language was thought to be the most likely reason for such findings. In addition, the majority of the nursing staff in the sample (74%) were Black females, the same ethnicity as the majority of their patients.

Language barriers between care providers and patients have often been reported as obstacles to the provision of culturally competent care [5, 17]. However, critical cultural competence (CCC) can also be affected by the ethnicity of both the healthcare providers and patients. Studies have shown that nurses working in a healthcare system where they make up the ethnic minority face racial discrimination [31] and fear from nurses who make up the ethnic majority. Additionally, many of their feelings may be devalued and they may lack equal opportunities for clinical skills development and career advancement [31–33]. Working in a hostile environment where skills are not developed can affect provider–patient interactions and competencies, thereby negatively influencing quality of care. Furthermore, when nurses in the ethnic majority have to deal with patients from an immigrant population or ethnic minority, they may face extensive communication difficulties [7, 34, 35]. One study reported that the majority of nurses felt that they lacked adequate cultural skills for dealing with these patient groups [35].

The concept of cultural competence and research in this area are more developed among the healthcare systems of Anglo-Saxon countries compared to Asian countries and the rest of the world [30]. This is unsurprising given the former countries have large multicultural communities and multiculturalism has been integrated into governmental policy since the early seventies [36]. North America leads the way in terms of being the first nation to integrate cultural diversity into the nursing curriculum in 1917 [37, 38]. Furthermore, Leninger (a US citizen) was a pioneer in developing transcultural nursing educational material, program development and academic instruction through PhDs in the subject. The American Association of Colleges of Nursing (AACN) has also provided strong support and endorsement of cultural competence education training and practice since the 1990s. Nurses who have received nursing education in these countries may be more aware of the concept and may have received training as part of their nursing degree curriculum, compared to nurses born and educated in Asian countries before moving to Canada. In fact, study teams looking at cultural competence conducted in Asian countries, such as Taiwan [26] and Japan [39], have noted the relative lack of understanding, familiarity and recognition of the concept of cultural competence within their own countries, which has led to a universal lack of knowledge regarding cultural competence. Furthermore, these countries are behind Anglo-Saxon countries in terms of formal cultural competency instruction, education, training and practice in healthcare [39].

## Conclusion and limitations

The results of this study indicate that age and country of birth can play a significant role in nurses’ perceptions of critical cultural competence. This study suggests that the nurses who were born in Canada and share a similar culture and language as their patients scored higher perceptions of cultural competence than those who were born in different countries. Thus, the results indicate that healthcare organizations with multicultural workforces or those that provide care to minority groups must provide ongoing cultural educational programs to increase the level of cultural competence among the nursing staff and to manage the difficulties that may arise due to cultural or linguistic differences.

However, this study has a number of limitations. The study’s participants were sampled from the contact details of registered nurses who expressed a willingness to participate in research studies. Therefore, the responses obtained from these participants may be different from those who chose not to participate, which may result in selection bias. However, the respondents were representative of the target population in terms of gender, educational level, mean of age, and experience in nursing profession [40]. Second, the current study was part of a much larger study, and the study instrument was distributed to participants in a pack along with five other instruments. The low response rate may be reflective of the significant length of time needed to complete all the survey instruments.

This study assessed the perception of critical cultural competence from the perspective of nurses; however, it would be beneficial if cultural competence level was explored with a larger heterogeneous sample that included registered nurses, educators and physicians. This would improve our understanding of how healthcare workers perceive their level of critical cultural competence, which would result in significant implications for policy and practice in such a multicultural environment. Qualitative research could also be conducted to understand the cultural competence of healthcare providers from patients’ perspectives, a topic that appears to be missing from the literature. There appear to be no studies in the literature assessing patient perspective of cultural competence of healthcare workers, but which might be valuable in creating nursing educational interventions to improve cultural competence and the basis for further research.

## Abbreviations

CCC: Critical cultural competence
CRNBC: College of Registered Nurses of British Columbia
